# Chronic brucellosis with sacroiliitis: A case report

**DOI:** 10.1002/ccr3.9525

**Published:** 2024-11-08

**Authors:** Bibek Shrestha, Pradeep Shrestha, Sudip Bastakoti, Prahlad Gupta, Shiva Ram Ale Magar

**Affiliations:** ^1^ Maharajgunj Medical Campus Institute of Medicine, Tribhuvan University Kathmandu Nepal; ^2^ Department of Internal Medicine Tribhuvan University Teaching Hospital Kathmandu Nepal

**Keywords:** Brucella, infection, spinal brucellosis, spine, spondylodiscitis

## Abstract

**Key Clinical Message:**

Brucellosis, especially with osteoarticular involvement, is uncommon and difficult to diagnose, but it should be considered in a case presenting with prolonged fever, joint pain, and back pain. The diagnosis requires a combination of history, serological examinations, and radiographic studies. When the condition is detected and treated early, the prognosis is good.

**Abstract:**

Brucella exploits host immune defenses to establish the chronic infection brucellosis, an endemic zoonotic disease. While human brucellosis affects multiple organs, osteo articular involvement is rare. We report the case of a 41‐year‐old woman with a history of goat farming who presented with fewer associated with low back pain and multiple joint pain for 4 months. Brucellosis was documented by positive serological tests (ELISA). Radiological findings suggested of right sacroiliitis and fracture line on the right sacral ala though had no history of trauma. Treatment consisted of NSAIDs and multiple antibiotic therapy. At follow‐up, her low back pain, joint pain, and fever had subsided. After 4 weeks of follow‐up, her symptoms were completely relieved with no recurrence. Brucellosis with atypical localization should be considered with a high index of suspicion based on detailed history and physical examination to ensure timely diagnosis and treatment.

## 1 INTRODUCTION

Brucellosis is an endemic zoonotic disease that affects wildlife. Its transmission to humans occurs through direct contact with infected animals or contaminated animal products. Brucella exploits host immune defenses to establish chronic infections which eventually leads to clinical manifestation ranging from fever, fatigue, joint pain to more severe complications such as endocarditis and neurological disorders.^1^ Human brucellosis may affect different organs, with symptoms varying in type and severity. This complexity tends to result in incorrect diagnosis. If left untreated, it could progress to the chronic stage and raise the possibility of disability.^2^

In the axial skeleton, brucellosis of the spine manifests itself in a variety of ways, including spondylitis, spondylodiscitis, pure discitis, sacroiliitis, and even facet joint infection.^3^ Sacroiliitis is an inflammation of the sacroiliac joints, often causing lower back pain that can extend to the legs.^4^ The etiology of sacroiliitis is complex, involving genetic, immunological, and environmental factors.^5^ Early diagnosis and its prompt treatment is essential in cases with spinal involvement to prevent further permanent neurological deficit. Doxycycline plus streptomycin is more effective than doxycycline plus rifampicin for treating brucellosis‐induced sacroiliitis.^6^ While the prognosis is generally favorable, relapses and sequelae like persistent joint pain can occur.^7^ In this report, we discuss the case of a 41‐year‐old woman diagnosed with Brucellosis complicated by right sacroiliitis.

## 2 CASE HISTORY/ EXAMINATION

A 41‐year‐old woman working as a goat herder presented to our hospital complaining of fever, low back pain, and other multiple joint pain for 4 months. The fever was intermittent, with episodes lasting 1–2 days followed by afebrile periods of days to a week. The fever reacted favorably to paracetamol, with a maximum documented temperature of 38.9°Celsius (102°Fahrenheit). She reported a low back pain along with bilateral pain of other joints including shoulder, elbow, wrist, and knee region. Morning stiffness was present for half to 1 h and joint pain was relieved by movement. She reported no history of trauma or injury.

On examination she was afebrile, blood pressure was normal (110/70 mmHg), pulse rate was normal (78 beats per minute), and SPO2 was adequate (96%). A neurological examination was found to be normal. Fabers' test and sacroiliac compression test of the right sacroiliac side were positive.

## 3 INVESTIGATIONS

With the history and examination, brucellosis, rheumatoid arthritis, spondyloarthritis, and tuberculous arthritis were kept for which different investigations were conducted. Upon investigation, her white blood cell count was 5800/cmm (normal 4000–11,000), hemoglobin was 11.3 gm% (normal: 12–18 gm%), PCV was 33.9% (normal: 37.5%–45%), platelets were 2,68,000/cumm (normal: 150000–400,000), ESR was 42 mm/h. (normal: 0–20 mm/h), HsCRP was 10.69 (levels >3 reflects active inflammation), and vitamin B12 was 481 pg/mL. Increased ESR and CRP levels suggests active inflammation of infectious origin. Serological tests (ELISA) for anti‐Brucella antibodies were positive with titer 1:160. The patient also underwent testing of ANA, RA factor, and HLAB27 typing, all of which were negative which ruled out rheumatoid arthritis, spondylarthritis. The Mantoux test for tuberculosis showed induration of 4 mm, indicating a negative result. The urinalysis and stool culture were normal.

**FIGURE 1:**
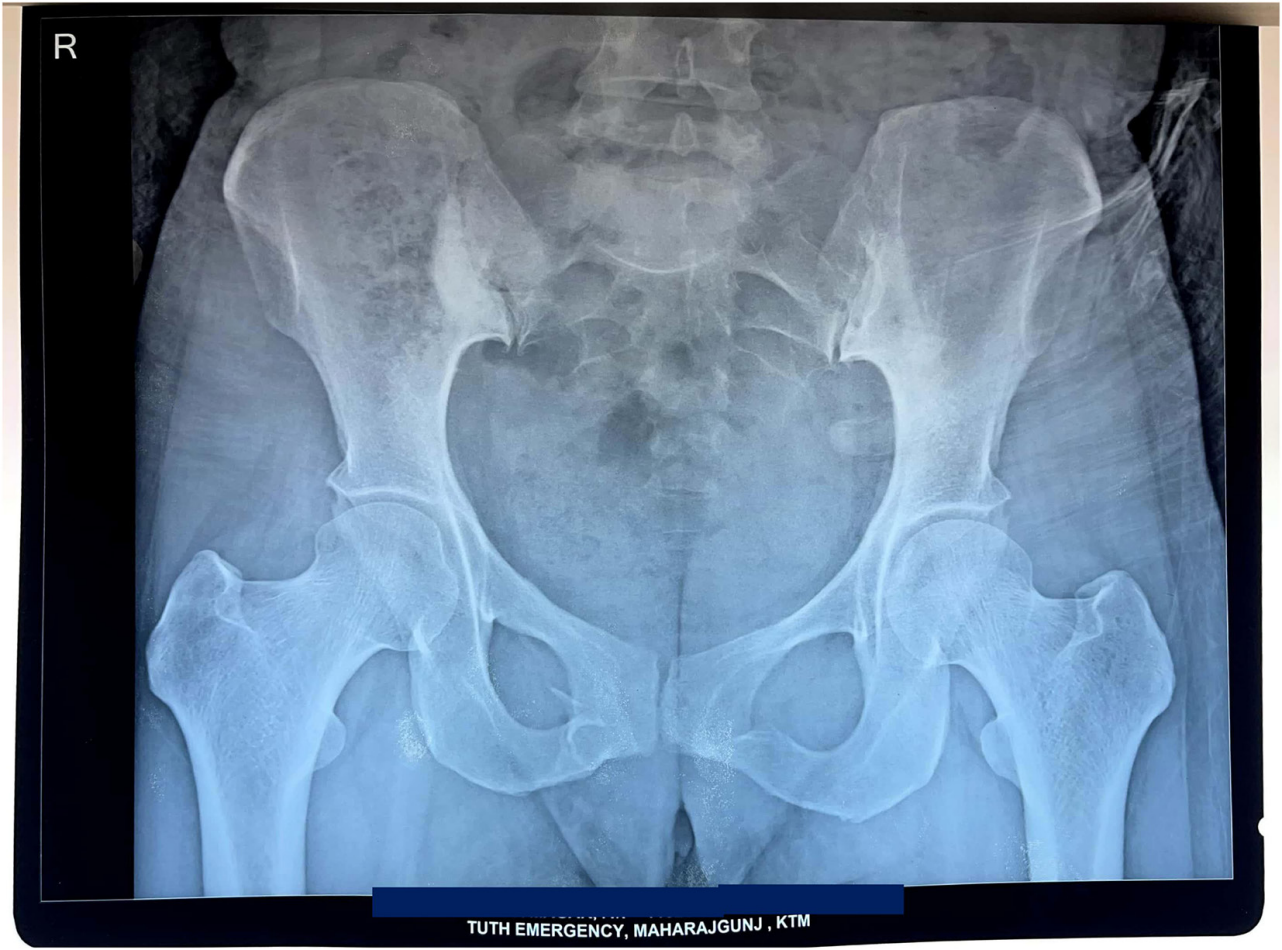
X ray of pelvis and lower spine. Sclerosis around right sacroiliac joint suggesting of right sacroiliitis.

**FIGURE 2:**
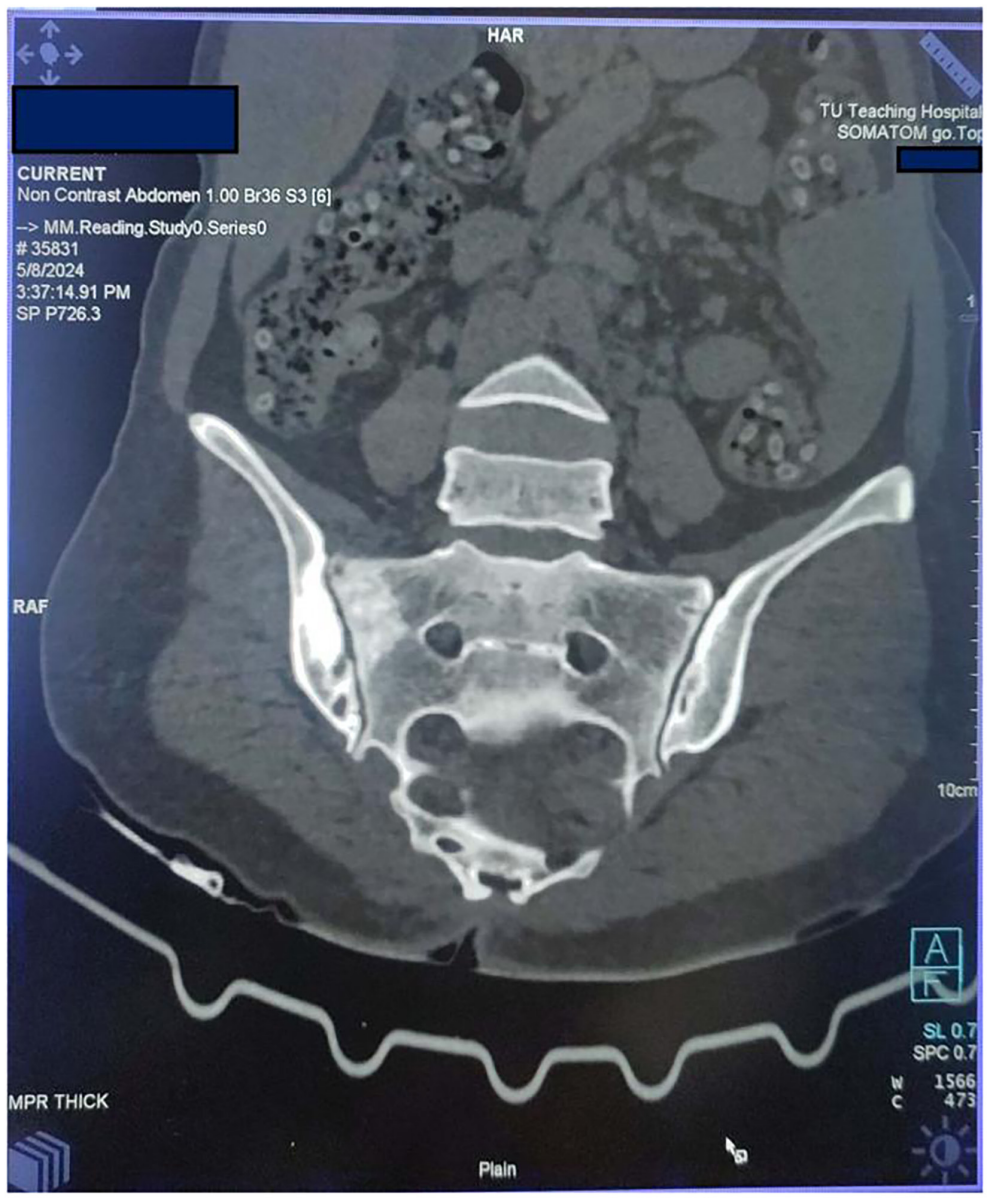
Axial bone window CT scan showing sacroiliitis of right sacroiliac joint.

X ray of pelvis and lower spine was done which revealed Sclerosis around right sacroiliac joint suggesting of right sacroiliitis. (Figure 1) Computed tomography scan of chest and abdomen revealed predominantly sclerotic changes in the articular surface of right sacroiliac joint, suggestive of right sacroiliitis, associated with a hypodense fracture line in the adjacent sacral ala. Axial bone window CT scan (Figure 2) revealed sclerosis and erosion around the right sacroiliac joint. Sacroiliac joint in post‐contrast images of MRI spine showed a subchondral low signal in the right iliac surface (likely sclerosis) and subchondral marrow enhancement in both the sacral and the iliac surface of the right sacroiliac joint, with enhancement of the adjacent tissue and patchy subchondral marrow oedema at the sacral aspect of the left sacroiliac joint.

## 4 CONCLUSION AND RESULTS

A diagnosis of brucellosis with sacroiliac involvement was made based on history, examination, serology, and radiological findings. The patient was treated with a combination of doxycycline (100 mg; twice a day), rifampicin (600 mg; once a day), and gentamicin (320 mg; IV). Following the treatment, the patient was closely observed. Her joint pain and low back pain slowly decreased, and fever was also subsided. She was discharged on the 18th day of her stay from hospital. She had followed up appointment at the outpatient department after 4 weeks of discharge and no fresh issues were identified.

## 5 DISCUSSION

Brucellosis is an endemic zoonotic disease present worldwide. It is transmitted by consumption of unpasteurized milk or raw, uncooked meat and by contact with affected animal products.^8^ The symptoms of brucellosis in its early stage may be nonspecific and mild, which makes diagnosis difficult and increases the risk of progression to spinal brucellosis. While clinical features and imaging findings can provide strong clues, the diagnosis of brucellosis can only be confirmed only by serological testing for Brucella spp. or isolation of Brucella spp. from blood, bone marrow, or other tissues.^9^ A literature review found skeletal involvement in 21.8% of acute cases, 34.7% of subacute cases, 25.7% of chronic cases, and 27.3% of relapsed cases, for a total of 25.3%.^10^ This review suggested that osteoarticular involvement is more likely in subacute, chronic and relapsed cases than in acute cases. In our case, patient had a chronic course of illness with history of fever and back pain for 4 months. In the acute phase, brucellosis presents with nonspecific symptoms such as undulating fever, weight loss, and weariness; in its chronic phase, months to years after the initial exposure, the musculoskeletal system can be involved, with the onset of back pain, arthralgias and sweating.^7^ The symptoms of our case and their duration over months are consistent with chronic brucellosis with spinal involvement. Clinical history, examination and investigation findings need to be correlated for the proper diagnosis of brucellosis. If brucella involves the axial skeleton, its manifestations may include spondylitis, spondylodiscitis, pure discitis, sacroiliitis, and even facet joint involvement.^6^ Typically, brucellosis presents with mild anemia, thrombocytopenia, relative lymphocytosis, leukopenia, or raised ESR and CRP levels in routine blood testing.^11^

In our case, elevated ESR, CRP level, and mild anemia were suggestive of chronic infection. Isolation and identification of Brucella spp., while necessary for the diagnosis, can be time‐consuming, requires specialized staff and is associated with safety risks. Therefore, serological testing is generally recommended.^12^ Serological testing for brucellosis is based on the identification of antibodies against either lipopolysaccharides or other bacterial antigens.^13^ Significant titers for diagnosis brucellosis in serological agglutination test is over 1:160; in endemic areas, above 1:320.^14^ In this case, the serological agglutination profile test was significant at a titer of 1:160 for two times at an interval of 4 weeks. Radiological assessment such as computed tomography (CT), and magnetic resonance imaging (MRI) can aid to identify spinal brucellosis. CT can identify vertebral degeneration and sclerosis, while MRI is sensitive for breakdown of vertebral body bone, intervertebral discs, and abscesses within and outside of spinal canal.^15^ In this case, CT showed predominantly sclerotic changes suggestive of pathology in the articular surface of the right sacroiliac joint, while MRI showed loss of cervical lordosis, disc desiccation at multiple levels, mild disc bulge at C5‐C6. The patient should be the primary consideration when selecting a suitable antibiotic combination. The WHO recommended a triple regimen of doxycycline (100 mg twice a day), rifampicin (600 mg/day), and streptomycin (1 g/day IM, 21 days) over 6 months as first‐choice regimen.^16^, ^17^ However, in our case, a combination of doxycycline (100 mg; twice a day), rifampicin (600 mg; once a day), and gentamicin (320 mg; IV, once a day) was used. Gentamicin have been used in this case due to easy availability and accessibility along with less side effect like ototoxicity and nephrotoxicity and being hospitalized gentamicin provides controlled dosing.

## AUTHOR CONTRIBUTIONS

**Bibek Shrestha:** Conceptualization; writing – original draft; writing – review and editing. **Pradeep Shrestha:** Conceptualization; writing – original draft; writing – review and editing. **Sudip Bastakoti:** Writing – review and editing. **Prahlad Gupta:** Conceptualization; writing – original draft; writing – review and editing. **Shiva Ram Ale Magar:** Conceptualization; writing – original draft; writing – review and editing.

## FUNDING INFORMATION

None.

## CONFLICT OF INTEREST STATEMENT

The authors have no conflict of interest to declare.

## ETHICS STATEMENT

The Institutional Review Board of the Institute of Medicine, Nepal, does not mandate ethical approval for the writing or publication of case reports, and patient consent was obtained. Informed written consent was obtained from the patient before writing this case report.

## CONSENT

Written informed consent was obtained from the patient to publish this report in accordance with the journal's patient consent policy.

## Data Availability

The datasets used and/or analyzed during the current study are available from the corresponding author upon reasonable request.
